# Preparation and Applications of Multifunctional MXene/Tussah Silk Fabric

**DOI:** 10.3390/ma18010169

**Published:** 2025-01-03

**Authors:** Bingbing Xu, Yue Zhang, Jia Li, Boxiang Wang, Yanhua Lu, Dehong Cheng

**Affiliations:** 1 College of Textiles and Garment, Liaodong University, Dandong 118003, China; xubingbing@liaodongu.edu.cn (B.X.); zhangyue19875@163.com (Y.Z.); lj18840597623@163.com (J.L.); bxwang0411@163.com (B.W.); yanhualu@aliyun.com (Y.L.); 2 Liaoning Provincial Key Laboratory of Functional Textile Materials, Liaodong University, Dandong 118003, China; 3 Key Laboratory of Jiangsu Province for Silk Engineering, Soochow University, Suzhou 215123, China

**Keywords:** MXene, tussah silk, conductivity, functional finishing, sensing

## Abstract

The development of functional textiles has become a key focus in recent years, aiming to meet the diverse requirements of modern society. MXene has excellent conductivity, hydrophilicity, and UV resistance, and is widely used in electromagnetic shielding, sensors, energy storage, and photothermal conversion. Tussah silk (TS) is a unique natural textile raw material and has a unique jewelry luster, natural luxury, and a smooth and comfortable feel. However, there are relatively few studies on the functional finishing of TS fabric with Ti_3_C_2_T_x_ MXene. Here, we developed a multifunctional MXene/tussah silk (MXene/TS) fabric by the deposition of Ti_3_C_2_T_x_ MXene sheets on the surface of TS fabric through a simple padding–drying–curing process. The obtained MXene/TS fabric (five cycles) exhibited excellent conductivity (4.8 S/m), air permeability (313.6 mm/s), ultraviolet resistance (ultraviolet protection factor, UPF = 186.3), photothermal conversion (temperature increase of 11 °C), and strain sensing. Thanks to these superior properties, the MXene/TS fabric has broad application prospects in motion monitoring, smart clothing, flexible wearables, and artificial intelligence.

## 1. Introduction

With the vigorous development of artificial intelligence and flexible electronics, smart wearables present remarkable application prospects. Smart wearables are inseparable from various flexible substrates, including polymer films, gels, paper, and textiles [1,2,3,4]. Textiles, as the medium that has the closest contact with the human body, process advantages such as flexibility, comfort, breathability, light weight, and close fit with the human body, thus emerging as one of the best carriers for smart wearables. The electronic fabric, obtained by integrating conductive materials onto textiles, and exhibiting excellent electrical conductivity, ultraviolet resistance, and photothermal conversion performance, has received extensive attention in intelligent sensing, health monitoring, electromagnetic shielding, healthcare, and multifunctional protection [5,6,7,8,9].

MXene, as an emerging 2D nanomaterial, has extensive applications in smart wearable textiles and is one of the ideal functional materials for textiles [10,11,12]. MXenes are transition metal (Ti, V, Cr, Nb, and Ta) carbides/nitrides with the universal formula M_n+1_X_n_T_x_ (*n* = 1, 2, and 3), where M represents an early transition metal (such as Ti, V, Cr, or Mo), X is carbon or nitride, and T_x_ is a surface functional group (–O, =O, and F). The unique atomic structure and electron transport properties endow MXenes with outstanding electrical conductivity, making them an ideal choice for energy storage systems and advanced electronic products [13,14,15,16,17]. MXenes show excellent hydrophilicity, facilitating their integration into various solutions and composite materials. Moreover, the mechanical strength of MXenes is exceptional, providing robustness and durability in smart wearables. The excellent electrical conductivity, abundant functional groups, hydrophilicity, unique dielectric properties, and high polarization anisotropy of MXenes make them suitable for textile modification. Ti_3_C_2_T_x_ is the most widely studied MXene at present. It is easy to synthesize and has broad application prospects in sensing, energy storage, catalysis, and electromagnetic shielding [18,19,20,21,22,23]. Polydiallyldimethylammonium chloride (PDDA) is a cationic polymer consisting of diallyldimethylammonium chloride monomer. It is positively charged and can bind tightly to negatively charged MXene through electrostatic interactions.

At present, many researchers try to treat fabrics with Ti_3_C_2_T_x_ MXene to obtain fabrics with different properties. For example, Zheng and co-researchers impregnated Ti_3_C_2_T_x_ on cotton fabric to prepare a pressure sensor. Taking advantage of the outstanding flexibility and three-dimensional porous structure of cotton fabric and the special sandwich architecture of the sensor, the Ti_3_C_2_T_x_/cotton fabric-based pressure sensor exhibited high sensitivity and a broad sensing range [24]. Zeng et al. designed a novel high-fire-safety cotton fabric with temperature-sensing, fire-warning, piezoresistivity, and Joule heating properties by coating MXene and carboxymethyl chitosan [25]. Pu et al. brush-coated MXene on one side of commercial polyamide fabric to develop a functional Janus fabric. The fabric makes full use of the dual characteristics of MXene’s ultra-low mid-infrared emission and high photothermal conversion ability, which simultaneously achieve radiant cooling and heating [23]. Shen et al. deposited a highly conductive MXene network onto a wood pulp fabric grid followed by hydrophobic methyltrimethoxysilane through a simple vacuum filtration method and sol–gel process, fabricating a multifunctional fabric with excellent electromagnetic shielding performance, hydrophobicity, and Joule heating performance [26]. However, there is a scarcity of research on the functional finishing of tussah silk fabrics with MXene. Tussah silk (TS) is a unique natural textile raw material, and it is also a protein fiber, like mulberry silk and wool. TS has superior healthcare features, remarkable moisture absorption, and remarkable permeability [27,28]. Additionally, it remains challenging to design and develop wearable TS textiles that simultaneously have the multifunctionality of breathability, ultraviolet resistance, photothermal conversion, and sensing.

Herein, we developed a multifunctional MXene/tussah silk (MXene/TS) fabric by the deposition of Ti_3_C_2_T_x_ MXene sheets on the surface of tussah silk fabric through a simple padding–drying–curing process. TS fabric possesses excellent flexibility and air permeability and is one of the suitable carriers for smart wearables. MXene is negatively charged and exhibits excellent conductivity and hydrophilicity, which can be nicely finished onto the TS fabric with positively charged cationic polymer polydiallyldimethylammonium chloride (PDDA). By integrating the advantages of MXene and TS fabric, the resultant MXene/TS fabric presents excellent functions such as conductivity, air permeability, ultraviolet resistance, photothermal conversion, and strain sensing, contributing to the development and application of MXene and new TS textile materials.

## 2. Materials and Methods

### 2.1. Materials

Titanium aluminum carbide (Ti_3_C_2_T_x_, 400 mesh) powder was purchased from 11 Technology. Lithium fluoride (LiF) was provided by Adamas (Shanghai, China). Hydrogen chloride (HCl, 37 wt%) was provided by Sinopharm Chemical Reagent Co., Ltd. (Shanghai, China). The tussah silk fabric was supplied by Dandong Huagao Trade Center (Dandong, China). Cationic polymer polydiallyldimethylammonium chloride (PDDA, 40 wt%) was purchased from Shandong Yousuo Chemical Technology Co., Ltd. (Linyi, China).

### 2.2. Synthesis of MXene

The delaminated MXene was synthesized according to the etching and delamination method with LiF/HCl as reported [29,30]. Typically, 1 g of LiF was added to 20 mL of 9 M HCl solution in a Teflon container and magnetic stirring was performed for 10 min. After LiF was completely dissolved, 1 g of Ti_3_AlC_2_ was added slowly and continuous magnetic stirring was performed at 50 °C for 24 h. The product was washed with deionized H_2_O via centrifugation (5 min per cycle at 5000 rpm) for multiple cycles until pH ≥ 6. The obtained multilayer Ti_3_C_2_T_x_ was sonicated in an ice bath for 60 min. Finally, a delaminated MXene supernatant was obtained by centrifuging at the speed of 3500 rpm for 30 min. The concentration of MXene dispersion was about 10 mg mL^−1^.

### 2.3. Preparation of MXene/TS Fabric

The tussah silk (TS) fabric was cut into a size of 5 cm × 10 cm, washed with detergent to remove surface impurities, and dried. The as-prepared TS fabric was immersed in a PDDA solution (0.1 wt%) for 20 min then dried. MXene/TS fabric was obtained by immersing PDDA-treated TS fabric in MXene dispersion using the process of two dips and two rolls–drying–curing. The influence of different process conditions on the fabric performance was discussed, including the finish cycles (1, 2, 3, 4, 5), dipping time (5, 10, 20, 30, 40 min), curing temperature (100, 110, 120, 130, 140 °C), and curing time (80, 100, 120, 140, 160 s).

### 2.4. Characterizations

The morphologies and microstructures of MXene and TS fabric were observed with a scanning electron microscope (SEM, JSM-IT100, JEOL Ltd., Tokyo, Japan). The UV–Vis spectra of MXene were obtained by a UV–Vis spectrophotometer (T6, Beijing Purkinje General Instrument Co., Ltd., Beijing, China). X-ray diffraction (XRD, D8 Advance, Bruker Corporation, Karlsruhe, Germany) measurements were performed at a scanning rate of 1° min^−1^ in the range of 3–70°. UV resistance was tested by a UV protection tester (UV-2000, Labsphere Inc., Sutton, NH, USA). The mechanical tests were conducted by an electronic universal testing machine (AGS-X, Shimadzu Corporation, Kyoto, Japan). The air permeability of the fabric was conducted by the fully automatic air permeability tester (YG461G, Ningbo textile instrument factory, Ningbo, China). The reflectance and K/S value of the fabric were measured by a spectrophotometer (CE7000A, Xrite Inc., Kentwood, MI, USA). The resistance changes of the MXene/TS fabric were tested with a digital source meter (Keithley DMM 2450, Tektronix Inc., Beaverton, OH, USA) and a multimeter (OWON B35T^+^, Fujian Lilipu Optoelectronics Technology Co., Ltd., Zhangzhou, China).

## 3. Results and Discussion

**Figure 1:**
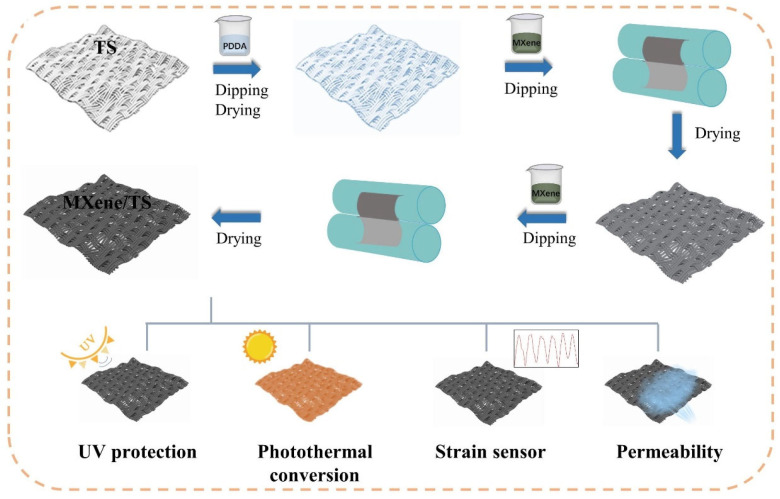
Schematic illustration of the fabrication process of the MXene/TS fabric.

Figure 1 demonstrates the fabrication process of the MXene/TS fabric. Firstly, the TS fabric from which impurities were removed was immersed in the cationic polymer PDDA solution and dried. Subsequently, MXene was finished onto the surface of the TS fabric through a simple padding–drying–curing process. Due to the difficulty of direct bonding between MXene and TS fabric, PDDA acts as a linker. The positively charged PDDA and negatively charged MXene are tightly bonded through electrostatic interactions.

**Figure 2:**
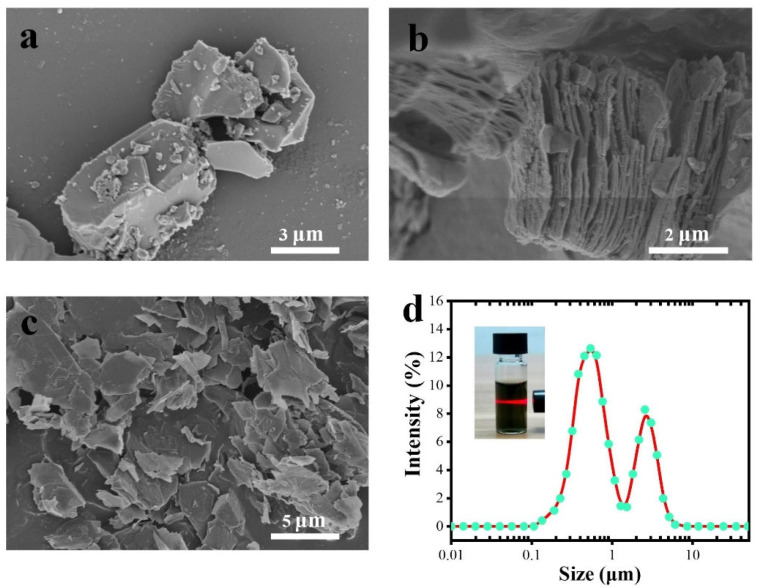
SEM image of (**a**) Ti_3_AlC_2_, (**b**) multilayer Ti_3_C_2_T_x_, and (**c**) delaminated Ti_3_C_2_T_x_ nanosheets. (**d**) Dynamic light scattering (DLS) size distribution of Ti_3_C_2_T_x_ nanosheets.

Figure 2 shows the preparation process of MXene nanosheets. Briefly, MXene nanosheets were obtained through selective etching Al atom layers from Ti_3_AlC_2_ with LiF/HCl solution, followed by sonication and centrifugation via a previously reported method [29]. The successful synthesis of delaminated MXene nanosheets was evidenced by the SEM image. As shown in Figure 2a–c, the Al atom layers of the densely packed Ti_3_AlC_2_ were removed to obtain accordion-like multilayered Ti_3_C_2_T_x_, and few-layer Ti_3_C_2_T_x_ nanosheets were exfoliated by sonication and centrifugation. Figure 2d shows the lateral size distribution of few-layer Ti_3_C_2_T_x_ is mainly concentrated at 0.5–3 μm. The inset shows a typical Tyndall effect for dark-green Ti_3_C_2_T_x_ aqueous dispersion, suggesting the formation of a stable and homogeneous aqueous dispersion of Ti_3_C_2_T_x_ MXene.

**Figure 3:**
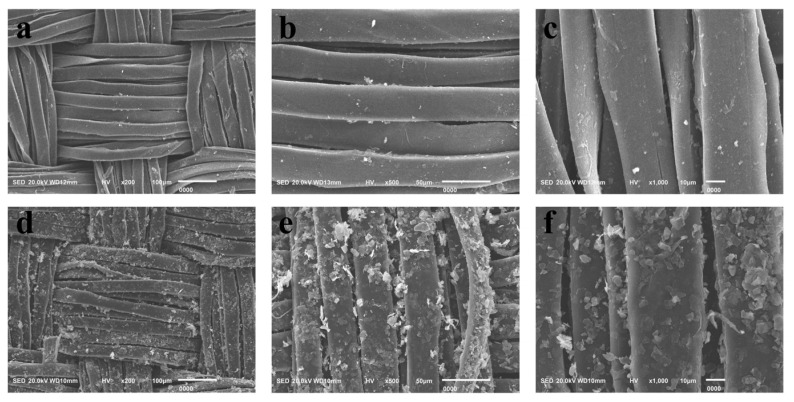
SEM images of (**a**–**c**) TS fabric and (**d**–**f**) MXene/TS fabric at different resolutions.

The morphologies and microstructures of TS fabric and MXene/TS fabric are characterized and shown in Figure 3. As shown in Figure 3a–c, the surface of the untreated TS fabric was smooth and free of impurities, while the surface of the treated MXene/TS fabric was covered with a large number of MXene nanosheets. Moreover, there was no obvious effect on the fabric structure and morphology after MXene was finished into the fabric. From the distribution of MXene on the single fiber, it can be seen that Ti_3_C_2_T_x_ MXene formed a dense conductive layer on the fiber surface. This proves that MXene was successfully finished onto the TS fabric.

**Figure 4:**
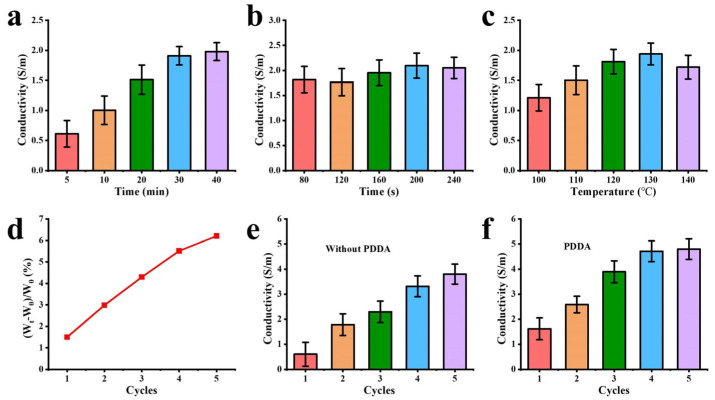
Conductivity of MXene/TS fabric at different (**a**) impregnation time, (**b**) curing time, (**c**) curing temperature, (**d**) cycles of two-dipping and two-rolling with PDDA, (**e**) cycles of two-dipping and two-rolling without PDDA. (**f**) Loading of MXene under different impregnation cycles.

To confirm the finishing process of tussah silk fabric with MXene, we investigated the electrical conductivity of MXene/TS fabric under different conditions. Firstly, with other conditions being the same (curing time of 200 s, curing temperature of 130 °C, two-dipping and two-padding), the impregnation time was adjusted to explore the change in electrical conductivity of MXene/TS fabric. As shown in Figure 4a, with the increase in impregnation time, the conductivity of MXene/TS fabric gradually increases, and when the impregnation time exceeds 30 min, the conductivity increases slowly. Figure 4b,c display the influence of curing time and curing temperature on the electrical conductivity of the MXene/TS fabric. Initially, the electrical conductivity of MXene/TS fabric enhanced with the prolongation of curing time and the elevation of curing temperature. This is attributed to the fact that the increase in curing time and temperature leads to the evaporation of water between the MXene nanosheets and the fabric, thereby augmenting the electrical conductivity of the fabric. MXene/TS fabric conductivity decreased when the curing time was extended to 240 s or the curing temperature exceeded 130 °C, because long curing time or high curing temperature increases the oxidation of MXene nanosheets.

Afterwards, we compared the effect of the presence of PDDA and impregnation cycles on MXene/TS fabric conductivity, as shown in Figure 4e,f. With or without the presence of PDDA, the fabric conductivity increased with the impregnation cycles. During the first three finishing cycles, the PDDA-introduced MXene/TS fabric exhibits a higher efficiency and conductivity in comparison to the MXene/TS fabric without PDDA. As the number of MXene finishing cycles increases, the difference in conductivity between the fabric with PDDA and that without PDDA becomes smaller. This is due to the fact that with more MXene being added, the MXene itself gradually forms a relatively continuous conductive network. Even without the assistance of PDDA, the large amount of MXene can provide a certain level of conductivity. In the initial stage, PDDA helps to better disperse and anchor MXene, enhancing the conductivity difference. But, as the MXene content becomes abundant and its own conductive channels are established, the promoting effect of PDDA on conductivity becomes less significant, resulting in a diminishing difference in conductivity between the two types of fabrics.

Compared with other fabrics, MXene/TS fabric not only exhibits high electrical conductivity, but also excellent dyeability, breathability and UV resistance (Table 1). 

**Figure 5:**
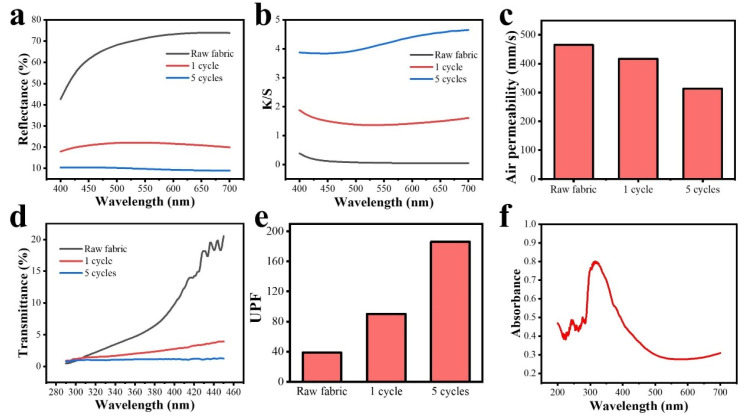
(**a**) Reflectance, (**b**) K/S value, (**c**) air permeability, (**d**) UV transmittance, and (**e**) UPF of fabric with different finishing times. (**f**) UV–Vis spectrum of Ti_3_C_2_T_x_ MXene.

As shown in Figure 5a,b, with the increase in finishing times, the reflectance of the MXene/TS fabric gradually decreased and the K/S value gradually increased, which further proves that MXene has been successfully finished on TS fabric. The measurement of air permeability is crucial in determining the breathability and overall comfort of a fabric, especially when considering its functional applications [35]. The TS fabric coated with MXene underwent rigorous testing to assess its air permeability. Figure 5c displays the air permeability of MXene/TS fabric with different finishing times. The air permeability of unfinished fabric is as high as 465.1 mm/s, the air permeability decreases to 416.8 after one finishing, and it stayed at 313.6 mm/s after five cycles. It is observed that the application of MXene onto the TS fabric affects its air permeability. Due to the nanoscale size and unique layered structure of MXene, it forms a thin, continuous layer over the surface of the TS fabric fibers. This layering potentially introduces additional barriers to airflow compared to uncoated fabric. However, it is essential to note that while there might be a decrease in permeability due to the coating, the fabric still maintains a level of breathability suitable for most functional applications. Moreover, the intrinsic properties of TS fabric, known for its lightweight and breathable characteristics, contribute positively to maintaining a balance between functionality and comfort.

In addition, the MXene/TS fabic exhibits excellent UV resistance. Figure 5d shows the UV transmittance of the original fabric is high, but after one finishing by MXene, the UV transmittance of the fabric is significantly decreased, indicating that the integration of MXene into TS fabric presents a significant enhancement in UV absorption properties. From the UPF values of fabrics with different finishing times, the UPF value of uncoated fabrics is 38.9. After one finishing, the UPF value of fabrics surges to 90.3, and it reaches 186.3 after five finishing times. (Figure 5e). This is primarily attributed to the inherent optical characteristics of MXene, which allows for the absorption of UV light across a broad spectrum. The two-dimensional structure of MXene provides an extensive surface area for the absorption of photons, making it an ideal candidate for enhancing UV protection in textiles (Figure 5f). Additionally, the deposition process ensures that the MXene flakes are uniformly distributed on the fabric surface, further maximizing its effectiveness in blocking UV rays. Furthermore, the synergistic effect between the TS and MXene results in a multifaceted approach towards UV resistance. While MXene acts as a physical barrier, absorbing and scattering UV light, the TS fibers contribute by reflecting a portion of the incident radiation. The combined mechanism offers superior protection compared to traditional textile materials or coatings alone.

**Figure 6:**
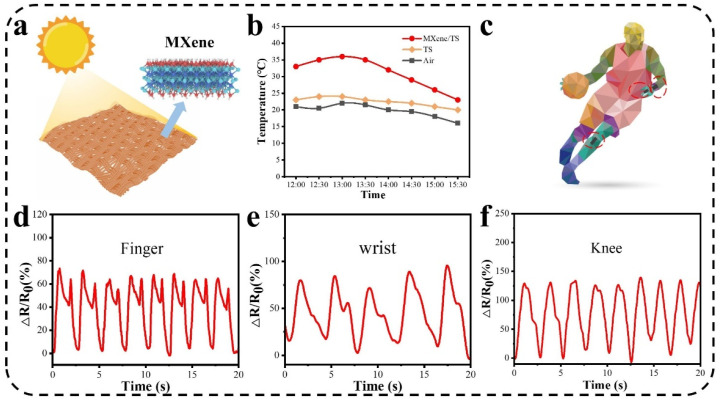
(**a**) Schematic diagram of photothermal conversion of MXene/TS fabric. (**b**) Photothermal conversion properties of TS and MXene/TS fabric. (**c**) Applications of the MXene/TS fabric strain sensor for various physiological signal detection. Relative resistance curves during (**d**) finger bending, (**e**) wrist bending, and (**f**) knee bending.

In the preliminary stages of investigating the photothermal conversion potential, a series of experiments were conducted to evaluate the performance of MXene/TS fabric. The experimental setup involved exposing the untreated and treated fabric to sunlight and measuring the temperature changes using an infrared thermometer. As shown in Figure 6a,b, the uncoated TS fabrics were exposed to sunlight and maintained a temperature of about 24 °C, while the fabrics coated with MXene reached a temperature of 35 °C, confirming the significant photothermal conversion efficiency of MXene/TS fabric. This is attributed to the excellent optical absorption properties of MXene, which effectively converts absorbed light into heat. MXene/TS fabrics also have excellent strain sensing properties, which are shown in Figure 6c–f as wearable strain sensors for real-time monitoring of human joint flexion and motion information. For example, MXene/TS fabric was attached to the finger, wrist, and knee of the tester for various motion monitoring. The relative resistance changes (RRCs) curves show different peak shapes depending on the amplitude of the movement. Moreover, the relative resistance changes correspondingly when the fingers, wrists, and knees are repeatedly bent or recovered, demonstrating excellent stability and repeatability of the MXene/TS fabric.

## 4. Conclusions

In summary, we successfully demonstrated the functional finishing and performance research of tussah silk fabric with two-dimensional transitional metal carbide Ti_3_C_2_T_x_ MXene. The deposition process of MXene on tussah silk fabric was carried out using a pad–dry–cure technique, which allowed for uniform coverage of the fabric surface. The resulting MXene/TS fabric (five cycles) exhibited excellent electrical conductivity (4.8 S/m), air permeability (313.6 mm/s), ultraviolet resistance (UPF = 186.3), and photothermal conversion potential (temperature increase of 11 °C). Furthermore, the MXene/TS fabric is used to detect various movement signals of the human body in real time, such as finger bending, wrist bending, and knee bending. The superior performance of the MXene/TS fabric shows a potential application in conductive fabrics, UV resistance, strain sensing, and photothermal conversion.

## Figures and Tables

**Table 1:** Comparison of the properties of MXene-based fabrics.

Materials	Conductivity	UPF	Air Permeability mm/s	Refs.
MXene@nonwoven	14.2 MΩ/sq~78 Ω/sq		237.7	[31]
MXene/cotton/spandex			91.85	[32]
MXene@silk	170 mS/cm	115		[33]
MXene/cotton	48 Ω/sq~5 Ω/sq	300	97	[34]
MXene@tussah silk	4.8 S/m	186.3	313.6	This work

## Data Availability

The original contributions presented in the study are included in the article. Further inquiries may be directed to the corresponding authors.
